# Supplementary material for: Patterns of Intron Gain and Loss in Fungi
Source: PLoS Biol. 2004 Nov 30;2(12):e422. doi: 10.1371/journal.pbio.0020422 (PMC532390; doi:10.1371/journal.pbio.0020422)
Supplement: Table S1 — Also available at http://genes.mit.edu/NielsenEtAl/. (4.3 MB ZIP). [file pbio.0020422.st001.zip › NielsenEtAl/html/967.html]

AN2514.1.NCU04596.1.MG01345.1.FG10036.1


```
 CLUSTAL W (1.82) Multiple Sequence Alignments - Introns Inserted


Sequence 1: NCU04596.1	333 aa
Sequence 2: MG01345.1	359 aa
Sequence 3: FG10036.1	312 aa
Sequence 4: AN2514.1	313 aa
Alignment Length: 376 aa
Number Identitical Residues: 107 aa
Alignment Score (without introns) 6120


MG01345.1 	MTKLVLSTG2NIVS-GGPSIIRKPGAFRSNLELTNSLRSNFLAAQEYYAAEAEAAAHN-N
NCU04596.1	MTKLILSTG2NIIS-GGPSIVRKPGA--SNLELVNSLKVSFQDAQLDYADEIENLNANGT
FG10036.1 	MTRLILSTA2NVML-AGPSIIRKPGNARSNLELVNSLRDNIAEAQRDYATDTEG------
AN2514.1  	-MKLVLSTS2NIMTSGGPSVIRQPSTFKSNVELINSLRANFQAAQQSIPSEPST------
          	  :*:***. *:: :.***::*:*.   **:** ***: .:  **   . : .       

MG01345.1 	NNNGNGAGIGNGNGNGNGINGDRRRVPASIWTTREKASLKRKAGNGDAAREPQQEQDEQG
NCU04596.1	TKTTTTTTNGTSNGVHEATNGD------SNTTDPVAGPLYWTA------------QDQKN
FG10036.1 	-------TNGFTN--DNALNGN------------HTAVELWTE--------------QQD
AN2514.1  	-----------------------------------ADSAAWTK--------------QED
          	                                         .               ::.

MG01345.1 	EVLFVPRIDWFAAGLQEEASQYDITAKLFFLP-TAPAASRR-RYAREALSLVLRELG-VR
NCU04596.1	NTIFVPRIDWETAALSEERSQYDITVKIFFLPTTTPDVSRRAQYTTEAMELVLKELGGIP
FG10036.1 	DALYVPRINWNAAGLREEPSQYEITVKFFVLS--GMSDKAREQHVKEALDLVRRELG-IT
AN2514.1  	DTLYIPAVDFSQRGLAEERSQYDITVKLFYLP--GIPVSRRCAHTKQAIDLVLKELH-VD
          	:.:::* :::   .* ** ***:**.*:* *.      . *  :. :*:.** :**  : 

MG01345.1 	AVDLLIVSLPGMSFEGDCEWEADKKNGEQG--------DDEEEAATWR-IVEDLHKEGLV
NCU04596.1	DVDLLVVSFPGMSFDGDCEWEADKLNASQG--------DDESEIATWQAAVEELHRQGLV
FG10036.1 	TIDLLIVSFPGISFEGSCEWEADKTNAHQG--------NLEEELATWK-VFESLHKQGLV
AN2514.1  	SIDLLIVSFPGIHFDAEDDSEEEESISVDGGSSFEHEDDFDSLVQTWK-CLESLHEQGKI
          	 :***:**:**: *:.. : * ::  . :*.:: . ..: :.   **:  .*.**.:* :

MG01345.1 	QRLGLSEFGSEKLARFLARKPTIRPAVDQINVKNCCSVPPPLAKLAKEQKIELLVHSDCT
NCU04596.1	KRLGISEFGTEKLARFIKR-VAIPPAVDQINLKNCCNVPPPLATLAKEQNVELLVHSDCT
FG10036.1 	KRLAVAEFGSEKLSAFIKH-TTVRPVIDQINLRNCCDVPPPLKKLAEDEGVELHVHNDCT
AN2514.1  	LQLGVAEFGSERLARFLEH-TRVKPAVNQINLKDCCVVPKSLILYAKEQKIDLLTHNDCN
          	 :*.::***:*:*: *: :   : *.::***:::** ** .*   *::: ::* .*.**.

MG01345.1 	DILPQGTLRELLGPGIRGAGILASESGGDDEEYEGGLLKGEIIPEWVIKYTAVVRDRGVI
NCU04596.1	DILPEGTLRELLGP--QGAGLLADPATGS------GGLTGNLTPQWVIKYTAVVKNRGVI
FG10036.1 	DILPHGTLRELLSHGSKGAGLLADSSNNG------SGLTGELVPEWVVRYTAFVQDRGVI
AN2514.1  	DILPKGTIRELLGPGERGAGILAPTPELDGK--DGGLVKGDIETQWVVKYTAVVKDRGVV
          	****.**:****. . :***:**  .  ...  ... :.*:: .:**::***.*::***:

MG01345.1 	ENKGYFAGAELRQT---
NCU04596.1	ENKGYFAGAELN-----
FG10036.1 	ENKGYFAGAELVRS---
AN2514.1  	ENKGYFALADVGSCVEA
          	******* *::    .:
```
